# Placement of EVD in pediatric posterior fossa tumors: safe and efficient or old-fashioned? The Vienna experience

**DOI:** 10.1007/s00381-023-05917-0

**Published:** 2023-03-23

**Authors:** Cora Hedrich, Johannes Gojo, Amedeo Azizi, Andreas Peyrl, Irene Slavc, Fabian Winter, Thomas Czech, Christian Dorfer

**Affiliations:** 1 Department of Pediatrics and Adolescent Medicine, Comprehensive Center for Pediatrics and Comprehensive Cancer Center, Medical University of Vienna, Vienna, Austria; 2 Department of Neurosurgery, Medical University of Vienna, Vienna, Austria

**Keywords:** Hydrocephalus, Posterior fossa tumors, EVD, Cerebrospinal fluid diversion, Pediatric neurosurgery, Pediatric neuro-oncology

## Abstract

**Purpose:**

The perioperative treatment of hydrocephalus in pediatric posterior fossa tumors with an external ventricular drain (EVD) is the treatment of choice in our center. We analyzed our experience in using EVD concerning safety and effectivity.

**Methods:**

This is a single-center retrospective cohort study of 100 consecutive pediatric patients who underwent resection for a newly diagnosed tumor in the posterior fossa between 2011 and 2022.

**Results:**

Of the 100 patients with posterior fossa tumors, 80 patients (80%) had radiological signs of hydrocephalus at presentation, 49 patients (49%) of whom underwent placement of an EVD. In 40 patients, the EVD was inserted at a mean of 2.25 days prior to the tumor resection; 9 had the EVD inserted during tumor resection (frontal trajectory in 7 patients, occipital trajectory in 2 patients). Histology revealed pilocytic astrocytoma in 48 patients, medulloblastoma in 32, ependymoma in 11, and other histologic entities in 9 patients. Gross total/near-total resection was achieved in 46 (95.83%) of the 48 pilocytic astrocytomas, 30 (93.75%) of the 32 medulloblastomas, and 11 (100%) of the 11 ependymomas. The mean number of total days with the EVD in place was 8.61 ± 3.82 (range 2–16 days). The mean number of days with an EVD after tumor resection was 6.35 ± 3.8 (range 0–16 days). EVD-associated complications were seen in 6 patients (12.24%) including one infection. None of these resulted in a worse clinical course or any long-term sequelae. Permanent CSF diversion at 6 months after surgery was necessary in 13 patients (13%), including two VP shunt, two SD-shunt, six endoscopic third ventriculostomy (ETV), and three combined VP shunt and ETV procedures. Patients with a medulloblastoma or ependymoma had a higher rate of permanent CSF diversion needed than the group of pilocytic astrocytoma patients (27.9% versus 2.13%, *p* < 0.001). In patients with metastatic disease, 7 of 17 patients (41.18%) needed a permanent CSF diversion, compared to 6 of 83 patients (7.23%) in the group without metastasis (*p* = 0.001).

**Conclusion:**

The treatment of hydrocephalus in pediatric posterior fossa tumors with an EVD as a temporary measure is safe and effective, provided that a multi-professional understanding for its handling is given and there is no need for a long transport of the children.

## Introduction

Brain tumors are the most common solid neoplasms in the pediatric population, and the majority of these tumors are located in the posterior fossa [1, 2]. Given their location, 73–100% of the children present with hydrocephalus that often requires treatment, before resection of the tumor may re-establish cerebrospinal fluid (CSF) circulation. Three different options in the treatment of CSF disturbances are available in this setting, including the placement of an external ventricular drain (EVD), upfront ventriculoperitoneal shunt insertion, and endoscopic third ventriculostomy (ETV). The discussion whether any of these methods may represent the safest and most efficient has been equally long as controversial. All these three methods have specific advantages and disadvantages, which to a not insignificant extent also depend on the framework in the individual centers.

At our institution, we have been following the concept to insert an EVD in case of symptomatic hydrocephalus and/or significant ventricular dilation before surgery and have not changed it over the last decades, as we felt comfortable and convinced by a perceived low rate of procedural problems.

With the advancement of endoscopic techniques over the last two decades, it seems that the discussion about the optimal perioperative treatment of the hydrocephalus has been directed towards ETV as the treatment of choice in many centers [3–7]. The Paris group was one of the first proponents of this technique [8]. In their series, the authors compared ETV to their former conventional protocol, which included administration of steroids, early surgical tumor resection, and EVD only if needed. The number of postoperative complications in the ETV group was significantly lower than in the conventional group being 25% versus 38%, respectively, and since their series pre-resection ETV has been adopted by many pediatric neurosurgery centers worldwide [8].

Now that endoscopic third ventriculostomy is also being increasingly discussed as the treatment of choice in multicenter clinical study protocols, we analyzed our experience with EVD insertion and raised the question if our concept is worth being continued or should be adapted.

## Methods

### Study design

After approval by the ethics committee of the Medical University of Vienna (EC 1358/2022), our database was screened to identify all children < 18 years of age who underwent a craniotomy for the resection of a newly diagnosed posterior fossa tumor between January 2011 and July 2022. The following patients were excluded: (1) patients with surgery for a tumor recurrence or biopsy only and (2) referrals from outside of Austria for adjuvant treatment only.

The following patient-specific data included in the study was gathered: sex, age at surgery, onset of symptoms, presence of hydrocephalus at time of presentation, EVD placement before tumor resection, EVD-related complications, local or disseminated disease at diagnosis, extent of resection, histopathology, and requirement for a permanent CSF diversion.

**Fig. 1:**
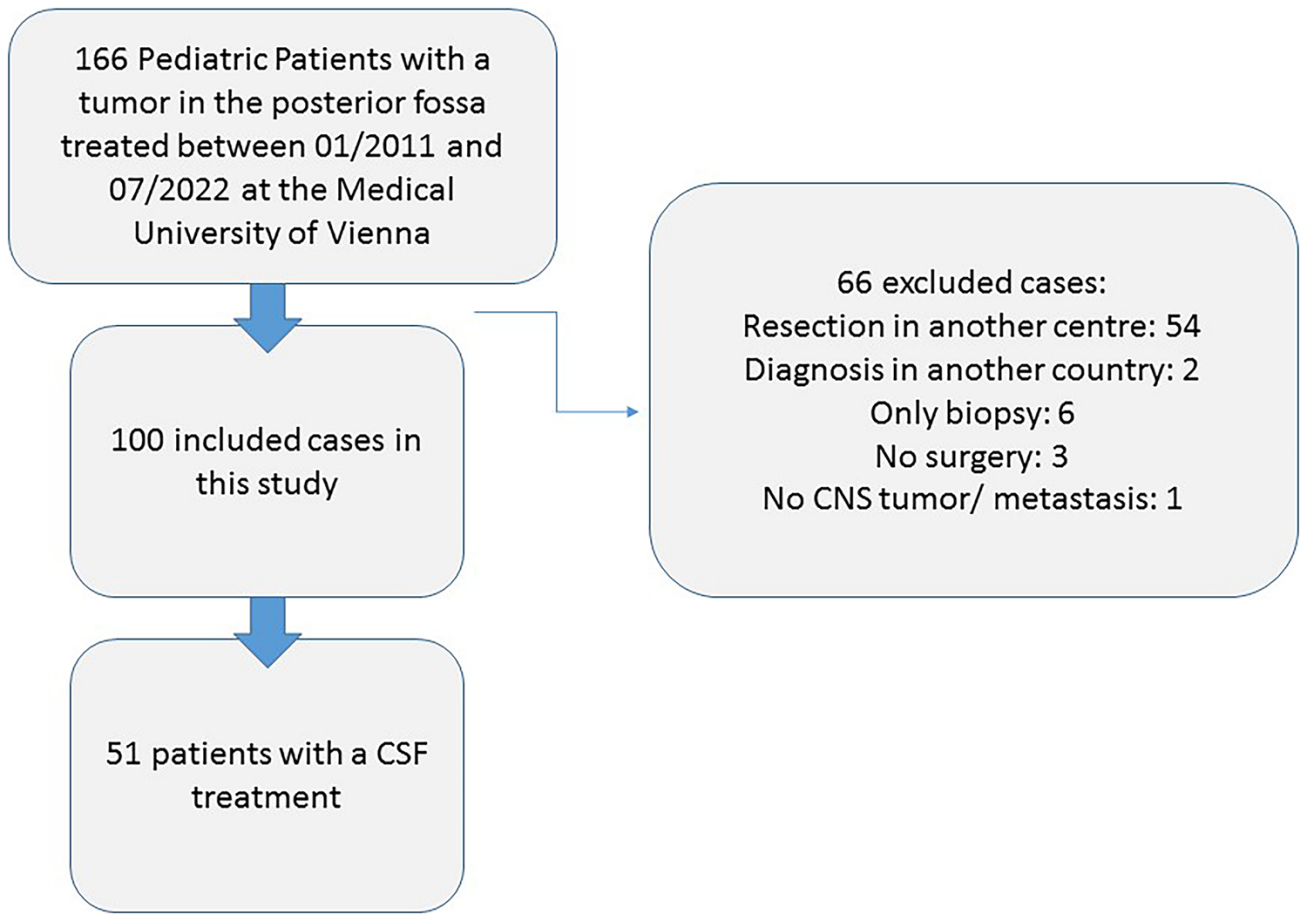
Flow diagram of patient screening, exclusion, and treatment pathway

According to the inclusion and exclusion criteria, 100 of 166 screened patients were included in this study. Fifty-four patients were excluded because they received their initial brain tumor surgery in another institution. We had to exclude two patients who received their initial diagnosis abroad and had a severe delay before receiving appropriate management of their brain tumor. Six patients were excluded because they only received a biopsy, three patients had no surgery, and one patient received resection of a brain metastasis of a kidney cancer. A patient flowchart is presented in Fig. 1.

### Institutional management of posterior fossa tumors and perioperative hydrocephalus

Children presenting with a posterior fossa tumor at our center are generally referred from peripheral centers or direct presentation in our clinic and all undergo a full tumor protocol MRI of the brain and spine at the earliest timepoint. All of them are either formally discussed in our weekly multidisciplinary tumor board or outside the formal board within the whole multidisciplinary team if the clinical condition warrants earlier surgery. In general, children were taken to the operating room (OR) at the earliest timepoint that allowed for the optimal operative environment usually within 2–3 days. Tumor resections were performed in a modified prone position (see Winter et al. [9]). No sitting position has been used in the time period of this study. In 72 patients (72%), a median suboccipital, in 23 patients (23%) a paramedian suboccipital, in three patients (3%) a retromastoidal, and in two patients (2%) an interhemispheric transtentorial approach was chosen.

The decision whether or not an EVD was inserted in case of enlarged ventricles on imaging at presentation was based on a case by case decision taking into account various factors such as clinical signs and symptoms, patient’s age, need for additional imaging, expected histology, and scheduled date of tumor resection. In general, our threshold was low to indicate placement of an EVD, as we felt this to be the safest way to avoid rapid deterioration and sequelae from decompensation, especially in very young children in whom neurologic monitoring is more difficult and the children needed to be sedated for the completion of the imaging work-up. In recent years, more antibiotic-impregnated catheters were used than in the past. Before tumor removal, the chamber of the EVD was set at a high level (between 10 and 20 cm above the level of the tragus depending on the age of the child) to ensure minimal CSF drainage necessary to treat a raised intracranial pressure, but at the same time avoid the risk of upward herniation through over-drainage. After surgery, the EVD was left in place until the incision of the tumor surgery was healed adequately with no signs of a pseudo-meningocele, CSF leak, or infection. For this purpose, special attention was given to the amount of CSF drainage. For the first days after surgery, the chamber of the EVD was usually kept at a low level (between 0 and 5 cm) and eventually increased depending on the clinical condition of the patient, the CSF morphology, the neurosurgeon’s impression of adequateness of dural closure, and the volume of drainage at the given level.

**Fig. 2:**
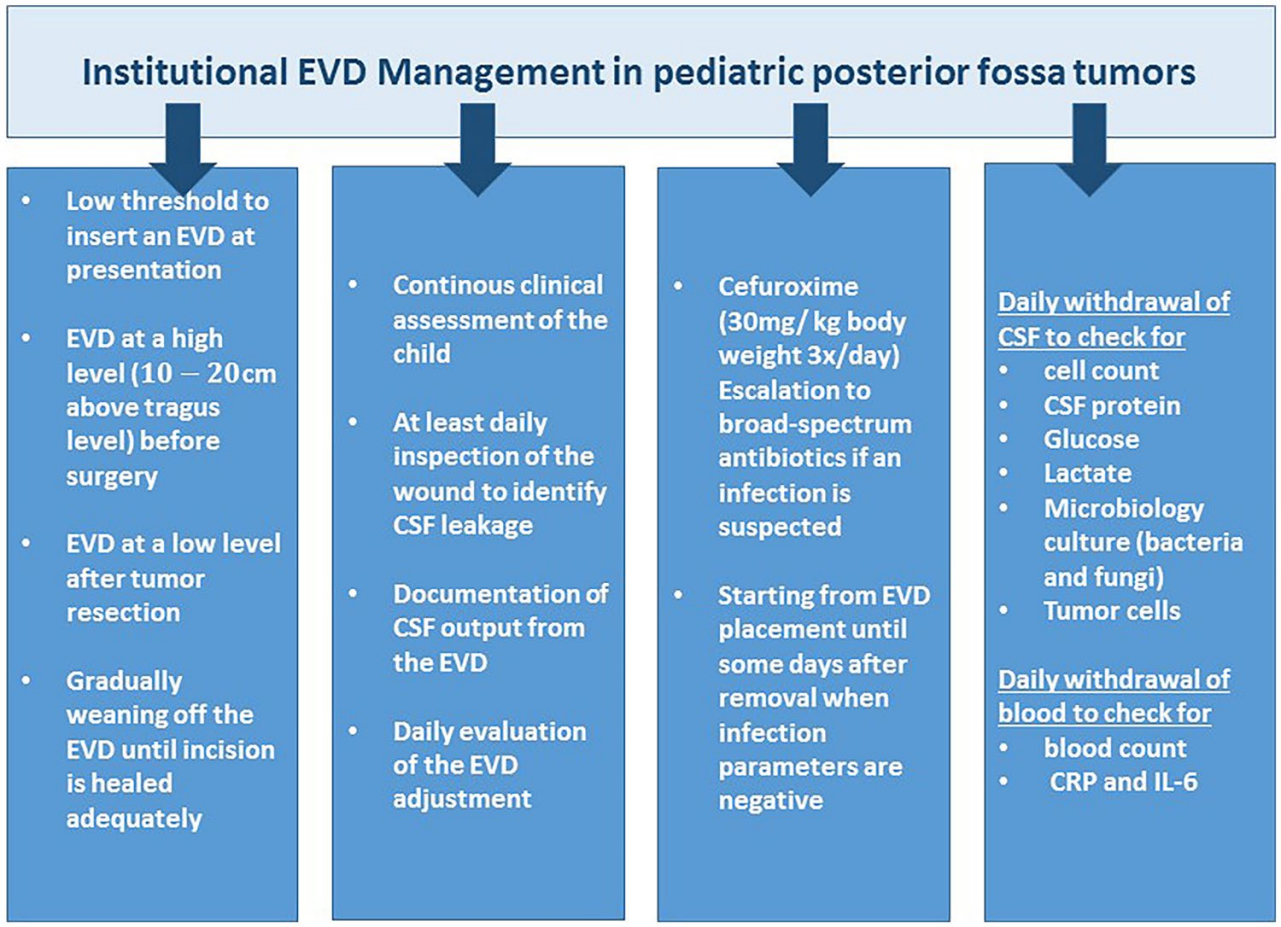
Institutional procedure for EVD management

Handling of the EVD on the pediatric ward was performed under meticulous hygienic conditions. All stopcocks were wrapped in sterile pads, disinfected before and after usage, and only manipulated with sterile gloves by trained neurosurgeons and pediatric neurooncologists. All children underwent prophylactic antibiotic treatment consisting of 30 mg/kg cefuroxime 3 × /day as long as the EVD was in place. In case of an allergy or other contraindications, an alternative antibiotic was administered. The CSF was analyzed daily for cell count, microbiology culture, and tumor cell pathology to enable instant identification of potential infection even before clinical symptoms appeared. A flowchart explaining the management of EVDs in our institution is presented in Fig. 2.

### Statistical analysis

Nominal variables were illustrated as percentages and total counts and compared with a chi-square test. If normally distributed, metric variables were described using means and standard deviation (SD). When more appropriate, in case of an uneven distribution, data was described as medians and interquartile ranges. Normally distributed, metric variables were analyzed by a *t*-test and skewed metric variables by a Mann–Whitney *U* test. The results were expressed by odds ratio (OR) with a 95% confidence interval (CI). *P*-values < 0.05 were considered statistically significant. The statistical analysis of this study was performed by using IBM® SPSS Statistics (Version 28.0 May 25, 2021) for Windows.

## Results

One-hundred patients (male to female ratio = 1:0.56) with a newly diagnosed tumor in the posterior fossa were included in this study. The mean age was 7.35 ± 4.17 years. Gross total resection/near-total resection was achieved in 46 (95.83%) of the 48 pilocytic astrocytomas, 30 (93,75%) of the 32 medulloblastomas, and 11 (100%) of the 11 ependymomas. Three patients (3%) with the histology of an ependymoma received a timely second look resection in which a gross total resection (GTR) could be achieved in two of the children and a near-total resection (NTR) in the third child. For these three patients, the extent of resection after the second surgery was used for the analysis of this study.

Clinical and demographic characteristics are detailed in Table 1.

**Table 1:** Patients and tumor characteristics

**Parameters**	***n* (%)**
**Sex**	
** Male**	64 (64%)
** Female**	36 (36%)
**Age**	Mean = 7.35 years; median = 7.11 years; SD = 4.17 years
	Minimum 4.8 months
	Maximum 17.8 years
**Histology**	
** Pilocytic Astrocytoma**	48 (48%)
** Medulloblastoma**	32 (32%)
** Ependymoma**	11 (11%)
Other Histology*	9 (9%)
**Metastasis at diagnosis**	
** M0**	83 (83%)
** M1**	5 (5%)
** M2**	5 (5%)
** M3**	7 (7%)
** M4**	0 (0%)
**Surgical procedure**	
** Partial resection**	5 (5%)
** Near-total resection**	21 (21%)
** Gross total resection**	74 (74%)
**Surgical approach**	
** Median suboccipital**	72 (72%)
** Paramedian suboccipital**	23 (23%)
** Retromastoidal**	3 (3%)
** Interhemispheric transtentorial**	2 (2%)

*(Diffuse astrocytomas *n* = 4, Ewing sarcoma *n* = 1, gliofibroma *n* = 1, hemangioblastoma *n* = 1, neuroendocrine tumor *n* = 1, vestibular schwannoma *n* = 1)

### Perioperative CSF management

At presentation, 80 patients (80%) had radiological signs of hydrocephalus. Forty-nine patients (49%) received an EVD before tumor removal. Forty were placed frontally as an individual procedure 2.25 ± 2.59 days before tumor resection and nine of them were inserted in the same procedure. Of these nine patients, the EVD was placed via a frontal burr hole in a supine position in seven and via an occipital burr hole in the modified prone position without a repositioning for the tumor resection in two patients. The mean number of days from diagnosis to tumor resection in patients with enlarged ventricles was 2.69 days. The mean total number of days with the EVD in place was 8.61 ± 3.82 (range 2–16 days). The mean number of days with an EVD in place after tumor removal was 6.35 ± 3.8 (range 0–16 days). All these characteristics are detailed in Table 2.

**Table 2:** Characteristics of EVD treatment

**Parameters**	***n* (%)**
**Radiological signs of hydrocephalus at time of diagnosis**	
**Yes**	80 (80%)
**No**	20 (20%)
**EVD before resection**	
**Yes**	49 (49%)
**No**	51 (51%)
**Days from CSF treatment to resection**	Mean = 2.25, median = 1, SD = 2.59 (range 0–14 days)
**Days from diagnosis to resection in the subgroup with hydrocephalus**	Mean = 2.69, SD = 3.33 (range 0–19 days)
**Days with EVD**	Mean = 8.61, median = 9, SD = 3.82 (range 2–16 days)

Two of those patients were treated by a combination of an EVD plus an endoscopic fenestration of a cyst, of whom one also had an ETV prior to the tumor resection. Another two patients did not receive an EVD, but an ETV and a tumor cyst puncture in one case each.

In 37 patients (75.5%), a conventional ventricular catheter (CODMAN^®^ HOLTER^®^ Ventricular Catheter, Integra Life Sciences) was inserted, and in 12 patients (24.5%), an antibiotic-impregnated catheter (CODMAN^®^ BACTISEAL^®^ Anti-Microbial Catheter, Integra Life Sciences) was used. There is a trend towards antibiotic-impregnated catheters, since eight of 17 patients (47.06%) received a BACTISEAL^®^ catheter between 2020 and 2022, compared to four of 32 patients (12.5%) before 2020.

### Postoperative complications related to hydrocephalus or EVD management

We encountered no complication such as EVD treatment failure, catheter malposition, dislocation, significant over- or underdrainage, and hemorrhage of any reason in our study cohort. No patient experienced a dangerous clinical scenario from an insufficiently treated hydrocephalus.

Only one patient had an CSF infection. This patient was a 2-year-old boy who presented in a severe clinical condition with signs of decompensating hydrocephalus. He was immediately taken to the OR for placement of the EVD and was subsequently monitored on the Pediatric Intensive Care Unit (PICU) for 6 days before he recovered to a level justifying tumor resection. Two days after tumor resection via median suboccipital approach, the EVD could be removed and the patient was further recovering. Two additional days later, however, a brisk CSF leakage from the former EVD outlet was encountered and a CT scan showed clear progressive formation of bilateral hemispheric hygromas. Consequently, a subdural drain was inserted and the CSF showed signs of infection being turbid with elevated cell count and protein levels (635 absolute cells/μl and protein levels of 276.8 mg/dl). He received a broad-spectrum antibiotic treatment consisting of combined vancomycin, meropenem, and gentamicin, under which the CSF normalized the following days. No germ could be cultivated in repeated microbiological and virological examinations of the CSF.

CSF leakage from the EVD outlet was observed in 6 patients (12.24% of patients with an EVD). One of these patients suffered from the CSF infection described above. However, in the other patients, the leakage was not associated with a worse clinical course, resulting complications or a delay in adjuvant therapy. The leakage resolved by itself in one patient, two patients received a secondary stitch in a bedside setting, and two patients received an ETV to treat persistent hydrocephalus.

### Permanent CSF diversion within 6 months after surgery (Table 3)

**Table 3:** Characteristics of post-resection hydrocephalus

**Parameters**	***n*** ** (%)**
CSF treatment after surgery	
Yes	13 (13%)
No	81 (81%)
Type of CSF treatment	
VP shunt	2 (2%)
ETV	6 (6%)
SD shunt	2 (2%)
VP shunt and ETV	3 (3%)

A permanent treatment for persistent hydrocephalus within 6 months after surgery was necessary in 13 patients (13%). This consisted of ventriculoperitoneal (VP) shunt placement in two patients (2%), ETV in six patients (6%), and three patients with a combined VP shunt and ETV procedure. Two patients (2%) received a subdural peritoneal (SD) shunt due to excessive postoperative hygroma formation. From the group of 49 patients who had an EVD perioperatively, 12 patients received a permanent CSF diversion (24.49%). In the group of 51 patients without perioperative EVD treatment, only one patient (1.97%) needed a VP shunt.

### Risk factors for need of permanent CSF treatment

Patients who had a medulloblastoma or ependymoma had a significantly higher risk to develop a need for permanent CSF diversion than the group of pilocytic astrocytoma patients (27.9% versus 2.13%, *p* =  < 0.001). In the group of patients who were metastasized (patients with M1–M3 status) at diagnosis, seven out of 17 patients (41.18%) needed a permanent CSF treatment compared to six out of 83 patients (7.23%) in the group of patients without metastasis (*p* = 0.001) (Table 4). Age at surgery and extent of resection did not correlate with the risk for permanent CSF diversion treatment.

**Table 4:** Univariate analysis of risk factors for need of permanent postoperative CSF treatment after 6 months

	**Permanent CSF treatment required after 6 months**		
	**Yes (13)**	**No (87)**	**Multivariate p Value**
**Radiological signs of hydrocephalus at diagnosis**			
Yes	13 (13%)	67 (67%)	0.044**
No	0 (0%)	20 (20%)	
**Histology**			
Pilocytic astrocytoma	2 (2%)	46 (46%)	< 0.001*
Medulloblastoma/ependymoma	17 (17%)	26 (26%)	
**Metastasis**			
M0	6 (6%)	77 (77%)	0.001**
Group of M1–M4	7 (7%)	10 (10%)	
**Extent of resection**			
Gross total/near-total	12 (12%)	83 (83%)	0.51 (NS)**
Partial	1 (1%)	4 (4%)	
**Mean age**	6.29 (SD 3.81)	7.5 (SD 4.23)	0.16 (NS)***
Age < 2 years	1 (1%)	6 (6%)	0.635 (NS)**
Age > 2 years	12 (12%)	81 (81%)	

*Chi-square test

**Fisher’s exact test

***t-test

## Discussion

Our study shows that the EVD-associated complication rate for the perioperative hydrocephalus management in children with posterior fossa tumors is very low (infection rate 2.04%, leak from the EVD outlet 12.24%, no cases (0%) of wound dehiscence), provided that a meticulous antiseptic handling of the system from the time of insertion until removal can be seamlessly achieved, and the whole involved multi-professional team has a good understanding about the aims, risks, and benefit of this management. We had no patient who suffered from a wound problem such as CSF leak, pseudo-meningocele, or wound breakdown that caused any delay in the start of further treatment.

In our experience, the insertion of an EVD is the most effective, safest, and easiest procedure to temporalize the CSF disturbance in this patient population. It is a well-standardized procedure that can be performed by any neurosurgeon and reduces the intracranial pressure instantly, and its maintenance can be directly monitored by visual inspection without the uncertainty given by the other temporizing procedures. In addition, a continuous drainage of CSF after surgery allows for removal of surgical debris and blood products and can be used to assist an uncomplicated healing of the incision. This is particularly important in malignant tumors as any delay in the start of adjuvant treatment can negatively impact the prognosis of the patient [10, 11].

Despite all these advantages of insertion of an EVD, ETV has become more popular in recent years. One of the main arguments against the treatment with an EVD was a presumed high risk of infection, which was reported to be as high as 32% [12]. While the reported rate of infection greatly varied among different centers [12–15], the definition of the underlying infection was very inconsistent being based on positive CSF cultures, clinical symptoms, or laboratory findings only. Even though we considered any of these criteria valid, we encountered only one single CSF infection (2.04%). Moreover, this patient presented in a poor clinical condition with herniating symptoms and would have undergone an EVD as an emergency procedure in all centers. In this patient, the EVD had to be left in place for 6 days before he recovered well enough to be eligible for a surgical tumor removal. He was successfully treated with antibiotics and does not suffer from any sequelae from the infection. Therefore, we could show that our EVD management protocol enables to reach an infection rate close to zero, refuting a high infection rate as a main argument against EVD placement.

This protocol includes daily CSF sampling and prophylactic intravenous antibiotic treatment, which can be also discussed under various aspects. CSF sampling is generally considered to increase the risk of infection; however, as the infection rate in our series is significantly lower than in those reports investigating this to be an independent risk factor, we can conclude that a meticulous sterile handling when sampling reduces this presumed risk [14]. Based on the results of our study, it can be discussed whether the daily CSF sampling in our patient cohort was exaggerated. It seems obvious that with a close to zero infection rate, it may not be necessary to take daily laborious measures to detect a possible infection at an early stage. Therefore, we need to evaluate the significance of daily CSF sampling in this specific patient cohort at our center and may change our current policy in the future. The administration of prophylactic antibiotics as in our protocol is evenly controversial, as this may cause resistance and side effects like Clostridium difficile colitis. However, even though the meta-analysis by Fried et al. [12] recommended to only give one dose of systemic antibiotics prior to EVD insertion, supported by some other prospective and retrospective case studies, as well as a few randomized trials on this question, none of these studies did investigate the very vulnerable patient population of pediatric posterior fossa tumors [16, 17].

Apart from infections, the rate of EVD-associated postoperative complications seems lower in our series compared to other series, reaching only 14.29% patients in the EVD group. For instance, in the series by Sainte-Rose et al., the CSF management-related complication rate in the groups treated by an ETV, by an EVD plus steroids only when deemed necessary, or without hydrocephalus at presentation was 25%, 38%, and 9%, respectively. In addition, procedural failures causing unsafe clinical scenarios leaving children with an insufficiently treated hydrocephalus did not occur in our series. In the series by Srinivasan et al., five out of 95 patients were considered treatment failures (5.2%). Two patients deteriorated after their ETV but prior to tumor resection, so their tumor resection had to be performed earlier than planned. This is a scenario that might lead to suboptimal OR conditions, meaning that not the planned OR room, surgeon, anesthetist, or technician necessary to perform intraoperative monitoring was available. In none of our patients, an earlier than planned operation was necessary because of an insufficiently treated CSF disturbance. Similarly, in none of our patients, we encountered a clinical deterioration caused by over-drainage and upward herniation, which has been reported as a risk and argument against the management with an EVD. In the series of Van Loon et al., two of 30 adult patients who received an EVD due to spontaneous cerebellar hemorrhage had the complication of an upward herniation [18].

Any delay of adjuvant treatment caused by a postoperative complication can have a negative impact on prognosis [10, 11]. Especially, CSF leakage and wound breakdown need to be avoided by all means. With our protocol that includes postoperative CSF drainage over some days to assist wound healing, we did not encounter any CSF leakage leading to a delay in treatment. In the series by Srinivasan et al., the rate of postoperative CSF leakage was 10.7% in the ETV group and 13.4% in the non-ETV group. No details are given on the impact of the delay of adjuvant treatment in these patients [3].

Apart from these arguments related to postoperative complications, some authors advocated that a preoperative ETV reduces the risk for the need of a permanent CSF diversion with a shunt. However, in our series, the rate of shunt dependency after surgery was 9.57%, which is lower compared to the rates reported in previous studies [19–21]. Compared to series using ETV as the treatment of choice, we encountered similar rates [3, 6, 8, 22]. Moreover, Srinivasan et al. recently showed that the use of an ETV before tumor resection did not prevent the need for postresection CSF diversion [3]. Based on their findings, they changed the practice at their institution again.

As discussed previously, the prerequisite for a safe and successful management of perioperative hydrocephalus with an EVD is not only based on a good multi-professional understanding of how to handle it, but also some basic structural conditions. At our institution, the Departments of Neurosurgery and Pediatrics are close to each other, basically door to door. This allows convenient short direct communication pathways, avoiding long walking distances or even the need to drive a car to another hospital, as it is the case in some centers. Moreover, the necessity for a long transport of children with an EVD in place is not without risks. These structural considerations need to be taken into account when discussing perioperative hydrocephalus management and might have contributed to the strategy in some centers [8].

## Conclusion

Even though the treatment of hydrocephalus in pediatric posterior fossa tumors with an EVD warrants a big multi-professional effort, we feel convinced that it is worth sticking to this management at our institution as it proofed to be safe and efficient.

## Data Availability

The datasets generated during the current study are available from the corresponding author on reasonable request.
